# Increased Oxidative Damage of RNA in Early-Stage Nephropathy in db/db Mice

**DOI:** 10.1155/2017/2353729

**Published:** 2017-10-19

**Authors:** Wan-Xia Wang, Shun-Bin Luo, Ping Jiang, Meng-Ming Xia, Ai-lian Hei, Yong-Hui Mao, Chuan-Bao Li, Guo-Xin Hu, Jian-Ping Cai

**Affiliations:** ^1^The MOH Key Laboratory of Geriatrics, Beijing Hospital, National Center of Gerontology, Beijing 100730, China; ^2^Department of Laboratory Medicine, Gansu Provincial Hospital, Lanzhou, Gansu 730000, China; ^3^Graduate School, Chinese Academy of Medical Sciences and Peking Union Medical College, Dongdan, Beijing 100730, China; ^4^Department of Clinical Pharmacy, The People's Hospital of Lishui, Lishui 323000, China; ^5^Department of Clinical Pharmacy, Ningbo Medical Center Lihuili Eastern Hospital, Ningbo 315000, China; ^6^Department of Nephrology, Beijing Hospital, Ministry of Health, Beijing 100730, China; ^7^Department of Laboratory Medicine, Beijing Hospital of the Ministry of Health, Beijing 100730, China; ^8^Department of Pharmacology, Wenzhou Medical University, University Town, Wenzhou, Zhejiang 325035, China

## Abstract

To evaluate RNA oxidation in the early stage of diabetic nephropathy, we applied an accurate method based on isotope dilution high-performance liquid chromatography-triple quadruple mass spectrometry to analyze the oxidatively generated guanine nucleosides in renal tissue and urine from db/db mice of different ages. We further investigated the relationship between these oxidative stress markers, microalbumin excretion, and histological changes. We found that the levels of 8-oxo-7,8-dihydroguanosine (8-oxoGuo) and 8-oxo-7,8-dihydro-2′-deoxyguanosine (8-oxodGuo) were increased in the urine and renal tissue of db/db mice and db/db mice with early symptoms of diabetic nephropathy suffered from more extensive oxidative damage than lean littermate control db/m mice. Importantly, in contrast to the findings in db/m mice, the 8-oxoGuo levels in the urine and renal tissue of db/db mice were higher than those of 8-oxodGuo at four weeks. These results indicate that RNA oxidation is more apparent than DNA oxidation in the early stage of diabetic nephropathy. RNA oxidation may provide new insight into the pathogenesis of diabetic nephropathy, and urinary 8-oxoGuo may represent a novel, noninvasive, and easily detected biomarker of diabetic kidney diseases if further study could clarify its source and confirm these results in a large population study.

## 1. Introduction

Diabetic kidney disease (DKD) is the leading cause of kidney failure worldwide and the strongest predictor of mortality in patients with diabetes [1, 2]. Oxidative stress caused by increased free radical production is believed to play a central role in the development of DKD [3]. Previous reports have indicated that DNA marker 8-oxo-7,8-dihydro-2′-deoxyguanosine (8-oxodGuo) oxidation is a useful clinical marker of diabetic nephropathy (DN) [4–7], but the conclusion is somewhat controversial [8].

In addition to DNA, RNA also undergoes significant oxidative damage. RNA oxidation is considered to be a marker of an early stage at which the clinical symptoms are very discrete in some diseases [9, 10] and may be useful for the prevention and therapy of these diseases. The RNA oxidation marker 8-oxo-7,8-dihydroguanosin (8-oxoGuo) was recently identified as an independent predictor of mortality in patients with established and treated type 2 diabetes [11]. Since DKD is the strongest predictor of mortality in patients with diabetes [1, 2], it is important to determine whether or not there is a relationship between RNA oxidation and diabetic nephropathy.

Our previous study evaluating nucleic acid oxidation in type 2 diabetes and exploring its role in the development of this disease revealed increased DNA and RNA oxidation in type 2 diabetes, and type 2 diabetes patients with complications exhibited higher levels of 8-oxoGuo than those without complications [12]. We also found that oxidative damage to nucleic acids in the kidney of diabetic rats was more obvious than in other organs, especially RNA oxidation [13]. Although we have proposed that 8-oxoGuo may be correlated with diabetic nephropathy and that 8-oxoGuo in urine could be a useful and sensitive marker of diabetic nephropathy, there is little direct information about whether urinary oxidative stress markers correlate with damage in the tissue of diabetic nephropathy or early changes of diabetic nephropathy.

The db/db mouse on the *C57BLKS* background has been investigated intensively and exhibits many features similar to human diabetic nephropathy. These mice are a good model for the early pathological changes of human DN [14, 15]. To confirm whether or not RNA oxidation is an early pathogenesis in DKD, we applied an accurate method based on isotope dilution high-performance liquid chromatography-triple quadruple mass spectrometry (ID-HPLC-MS/MS), a sensitive and reliable method, to evaluate oxidative guanine nucleosides in renal tissue and urine from db/db mice of different ages. We further investigated the relationship between the urinary excretion of oxidative stress markers, microalbumin excretion, and histological changes. Our results suggest that urinary 8-oxoGuo could be a novel biomarker of diabetic kidney diseases.

## 2. Materials and Methods

### 2.1. Chemicals

The 8-oxodGuo (>98% purity), 2-deoxyguanosine (dGuo; >98% purity), guanosine (Guo; 98% purity), deferoxamine mesylate (DFOM), and HPLC-grade methanol were obtained from Sigma-Aldrich Inc., USA. 8-oxoGuo (>98% purity) was obtained from Alexis Biochemicals (San Diego, CA, USA). HPLC-grade ammonium acetate was obtained from Fisher Scientific, USA. Heavy-isotope-labeled 8-oxo-[^15^N_5_]dGuo, [^15^N_5_]dGuo, and [^15^N_5_]Guo were obtained from Cambridge Isotope Laboratories (Andover, MA, USA), and 8-oxo-[^15^N_2_^13^C_1_]Guo was obtained from Toronto Research Chemicals (Toronto, Canada). Water was deionized at 18.2 MΩ.

### 2.2. Experimental Animals

BKS.Cg-Dock7^m^ +/+ Lepr^db^/JNju (db/db) mice were used as a model of type 2 diabetic nephropathy [14, 15], and their lean littermates (db/m) were used as controls. Male mice were purchased from Nanjing Biomedical Research Institute of Nanjing University and maintained under specific-pathogen-free conditions (24 ± 2°C, 12 h light/dark cycle with light on at 7:00 AM) with free access to water and food. Db/db mice and their lean littermates (db/m) were divided into four age groups (ages 4, 8, 12, and 16 weeks), with each group including six mice. This study was performed with the approval of the local ethics committee, and all of the experiments were carried out according to the *National Institutes of Health Guide for the Care and Use of Laboratory Animals.*

### 2.3. Sample Collection

For the measurement of fasting blood glucose, blood was obtained from the tail vein after fasting animals for 12 h and analyzed with a portable blood glucose monitor (Bayer's BREEZE 2 blood glucose meter, Germany). Well-mixed 24 h urine was collected from mice in metabolic cages, and after it was centrifuged at 3000*g* for 10 min, supernatant was frozen at −80°C until use. Blood was collected into a Na_2_EDTA anticoagulant-pretreated vacuum tube after mice had fasted for 16 h and was immediately centrifuged at 4°C, 3000 rpm for 10 min. Plasma samples were isolated and frozen at −80°C before the analysis. After being dissected on ice, the kidneys were frozen in liquid nitrogen immediately and stored at− 80°C or placed in fixatives according to the experimental design.

### 2.4. Assessment of Renal Function and Microalbuminuria

A high-performance liquid chromatography with UV detector system (HPLC-UV) method developed by our laboratory [16] was used for creatinine quantitation in plasma and urine. Urinary microalbumin excretion was measured using a specific enzyme-linked immunosorbent assay (ELISA) for the quantitative determination of microalbumin in mouse urine (ab108792; Abcam, Cambridge, UK).

### 2.5. Histopathology

Kidneys were immersed in 10% formalin and embedded in paraffin. Sections (5 *μ*m thick) were processed for the histopathology study. The histopathology was investigated using hematoxylin and eosin (H&E) and periodic acid-Schiff (PAS) staining. In addition, small pieces of renal cortex were fixed in 2% glutaraldehyde, postfixed in 1% osmium tetroxide, and embedded in Araldite M (Sigma-Aldrich). Ultra-thin sections were counterstained with uranyl acetate and lead citrate and examined using a transmission electron microscope. Electron micrographs were used to determine the glomerular basement membrane (GBM) thickness and podocyte foot process width (FPw) as previously described [17, 18].

### 2.6. Preparation and Hydrolysis of Nucleic Acids from the Kidney

The preparation and hydrolysis of nucleic acids were performed according to our laboratory protocol with some modifications [16]. In brief, genomic DNA was prepared using the ESCODD recommended protocol [19]. Total RNA was extracted using the TRIzol (Invitrogen, CA, USA) reagent according to the manufacturer's instructions. DFOM was added to reduce the background levels of oxidized DNA and RNA.

For DNA hydrolysis, aliquots of samples containing 20 *μ*g of nucleic acids were subjected to denaturation by heating at 95°C for 3 min and chilling rapidly. The denatured DNA was then incubated with nuclease P1 (Wako Pure Chemical, Osaka, Japan) in 0.3 M sodium acetate-1 mM ZnSO4, pH 5.3, and incubated at 37°C for 2 h. Five units of alkaline phosphatase (New England Biolabs, USA) was then added, and the mixture was incubated for 1 h at 37°C. To 90 *μ*l of the DNA hydrolysate solution, 5 *μ*l of 100 ng/ml 8-oxo-[^15^N_5_]dGuo, and 5 *μ*l of 10 *μ*g/ml [^15^N_5_]dGuo were added. The samples were then gently mixed and centrifuged at 12,000*g* for 5 min at 4°C, and aliquots of the supernatant were taken for the HPLC-MS/MS analysis.

For RNA hydrolysis, 20 *μ*g of RNA in 80 *μ*l of 1 mM DFOM was incubated with 5 units of nuclease P1 at 37°C for 2 h, followed by an additional incubation with 5 units of alkaline phosphatase at 37°C for 1 h. Then, 5 *μ*l of 100 ng/ml 8-oxo-[^15^N_2_^13^C_1_]Guo and 5 *μ*l of 5 *μ*g/ml [^15^N_5_]Guo were added to 90 *μ*l of the RNA hydrolysates. These samples were also gently mixed and centrifuged at 12,000*g* for 5 min at 4°C, and aliquots of the supernatant were taken for the HPLC-MS/MS analysis.

### 2.7. Preparation of Urine Samples for the Oxidatively Generated Guanine Nucleoside Analysis

Frozen urine was thawed at 37°C for 5 min and centrifuged at 12,000*g* for 5 min at 4°C. For the oxidatively generated guanine nucleoside analysis, 100 *μ*l of urine was mixed with 400 *μ*l of 70% methanol solution to obtain a 1 : 5 diluted urine sample. Then, 10 *μ*l of 100 pg/*μ*l [^15^N_5_]8-oxodGuo and 10 *μ*l of 100 pg/*μ*l [^15^N_2_^13^C_1_]8-oxoGuo were added to the urine sample and centrifuged at 12,000*g* for 15 min at 4°C. Finally, 100 *μ*l of the supernatant was collected for the HPLC-MS/MS analysis of 8-oxodGuo and 8-oxoGuo.

### 2.8. Chromatographic and Mass Spectrometric Analysis

An Agilent 1290 Infinity UHPLC system was used to directly inject 4 *μ*l samples onto an Agilent Zorbax SB-Aq (1.8 *μ*m, 3.0 × 100 mm) column that was maintained at a column temperature of 50°C. Sample separations were achieved using a 300 *μ*l/min flow rate with the mobile phase consisting of 10 mM ammonium acetate at pH 3.75 (A) and 100% methanol (B). The sample room temperature was kept at 4°C. Early- and late-eluting components were discarded to reduce the contamination of the ion source.

An Agilent 6490 QQQ LC/MS system with a high-flow iFunnel technology ionization source controlled by Agilent MassHunter Workstation software program (version B.04.01; Agilent) was used for all HPLC-MS/MS sample analyses. All acquisition methods used the following parameters: 3500 V capillary voltage and 1500 V nozzle voltage, a sheath gas flow of 11 L/min (UHP nitrogen) at a temperature of 400°C, a drying gas flow of 14 L/min at a temperature of 200°C, and nebulizer gas flow at 20 psi. Quantification was performed using a multiple reaction monitoring model with a dwell time of 100 ms per transition for all compounds. All of the optimized values are summarized in Supplementary Table 1s to 3s available online at https://doi.org/10.1155/2017/2353729. The data processing was carried out using the MassHunter Workstation software program for Quantitative Analysis (version B.06.00; Agilent).

### 2.9. Statistical Analysis

All values are presented as the mean ± standard deviation (SD). Statistical significance was determined by a one-way analysis of variance (ANOVA), and levels of 8-oxoGuo or 8-oxodGuo between groups were compared by an ANOVA with the post hoc least significance difference test. A Pearson's correlation analysis was performed to examine the relationships between nucleotide acid oxidation markers and urinary microalbumin. Multiple linear regression analysis was further used to determine the relationships between nucleotide acid oxidation markers and urinary microalbumin. Statistical analysis was performed using the SPSS 22.0 software program (IBM Corporation, New York, NY, USA), and graphs were plotted using the GraphPad Prism 5.0 software program (GraphPad Software Inc., La Jolla, CA, USA). A two-sided *P* value < 0.05 was deemed statistically significant.

## 3. Result

### 3.1. Physical and Biochemical Characteristics of the Study Subjects

**Figure 1:**
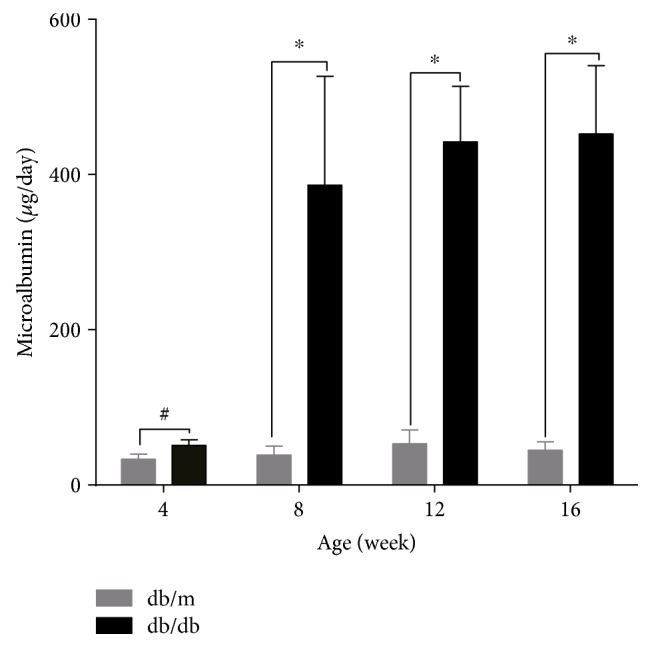
Increased albuminuria in db/db mice. db/db represents BKS.Cg-Dock7^m^ +/+ Lepr^db^/JNju mice and db/m represents their lean littermates. Samples from 4-, 8-, 12-, and 16-week-old mice were examined. From 8 to 16 weeks of age, db/db mice showed increased microalbuminuria compared with their lean db/m littermates. The data are shown as the means ± SD. (white-colored bars) db/m. (black-colored bars) db/db, ^∗^*P* < 0.05 versus respective control, ^#^*P* > 0.05 compared with the control.

**Figure 2:**
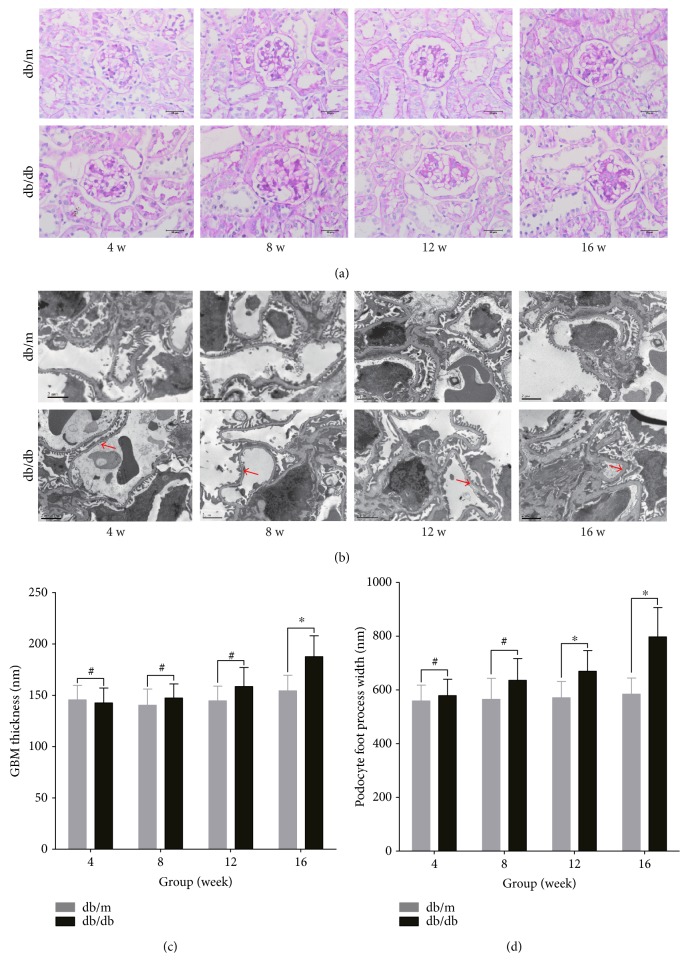
Histological and ultra-structural characteristics in diabetic db/db mice. db/db represents BKS.Cg-Dock7^m^ +/+ Lepr^db^/JNju mice, and db/m represents their lean littermates. Samples from 4-, 8-, 12-, and 16-week-old mice were examined, and the results are shown as 4 w, 8 w, 12 w, and 16 w, respectively. (a) Periodic acid-Schiff stain (original magnification, ×400) indicated a normal appearance in the outer cortex of control db/m mice and an increase in the mesangial matrix and glomerular hypertrophy in diabetic db/db mice. Scale bar: 25 *μ*m. (b) An electron-microscopic image (original magnification, 8000x) shows a normal appearance of the glomerulus in db/m mice and podocyte effacement or loss and mesangial extracellular matrix expansion and irregular glomerular basement membrane (GBM) thickness (red arrow) in db/db mice. Scale bar: 2 *μ*m. (c) GBM thickness (nanometers). (d) Podocyte foot process width (FPw) (nanometer). The GBM and FPw are shown as the means ± SD. (white-colored bars) db/m. (black-colored bars) db/db, ^∗^*P* < 0.05 versus respective control, ^#^*P* > 0.05 compared with the respective controls.

The body weight, kidney weight, and blood glucose were significantly higher for the db/db mice than for the db/m mice (Table 1). Total kidney-to-body weight ratios were lower in all db/db mice than in db/m mice. Although the difference in plasma creatinine was not statistically significant between the experimental groups, increased microalbuminuria (Figure 1) was shown in some groups of db/db mice (8- to 16-week-old mice) compared to the same-age db/m mice. Renal histology in these groups of db/db mice (8- to 16-week-old mice) was characterized by mesangial extracellular matrix expansion (Figure 2(a)). The glomerular ultrastructure was examined by transmission electron microscopy (Figure 2(b)). Compared with the control mice, there was segmental thickening of the GBM in db/db mice (8- to 16-week-old mice) with no electron-dense deposits (Figures 2(b) and 2(c)). In addition, podocyte effacement or loss and mesangial extracellular matrix expansion were more apparent in db/db mice (4- to 16-week-old mice) than in the same-age db/m mice (Figures 2(b) and 2(d)). Taken together, these findings show that the renal changes in db/db mice were consistent with early diabetic nephropathy.

### 3.2. Quantitation of Oxidized Guanine Nucleosides from DNA or RNA of the Kidney

**Figure 3:**
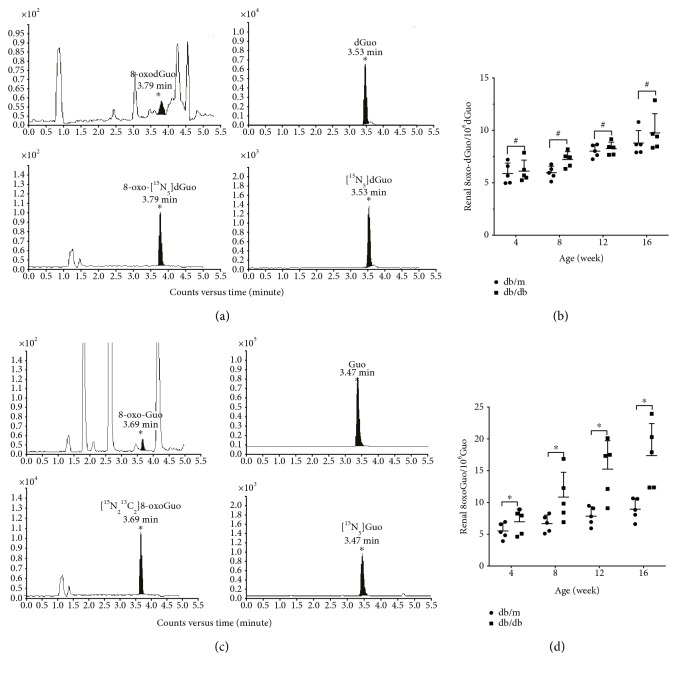
The HPLC–MS/MS determination of the concentrations of 8-oxodGuo and 8-oxoGuo in the kidneys of db/db mice. db/db represents BKS.Cg-Dock7^m^ +/+ Lepr^db^/JNju mice, and db/m represents their lean littermates. Samples from 4-, 8-, 12-, and 16-week-old mice were examined. (a) HPLC-MS/MS chromatograms of 8-oxodGuo, dGuo, 8-oxo--[^15^N_5_]dGuo, and [^15^N_5_] dGuo. (b) Increases in the level of 8-oxodGuo/10^6^ dGuo in the kidneys of db/db and db/m mice (*n* = 5 per group). (c) HPLC-MS/MS chromatograms of 8-oxoGuo, Guo, 8-oxo-[^15^N_2_^13^C_1_]Guo, and [^15^N_5_]Guo. (d) Increases in the level of 8-oxoGuo/10^6^ Guo in the kidneys of db/db and db/m mice (*n* = 5 per group). The data are shown as the means ± SD. (black-colored dots) db/m. (black-colored squares) db/db, ^∗^*P* < 0.05 versus the respective controls. ^#^*P* > 0.05 compared with the respective controls.

We adopted the ESCODD-recommended protocol and further applied deferoxamine methylate to reduce the background level of oxidation. DNA was hydrolyzed to nucleosides by successive treatments with nuclease P1 and alkaline phosphatase. To each sample, [^15^N_5_] dGuo and 8-oxo-[^15^N_5_]dGuo were added to provide appropriate internal standards. The mixtures were subsequently applied to the HPLC-MS/MS system for the quantification of the two types of deoxyguanosine: dGuo and 8-oxodGuo. As shown in Figure 3(a), dGuo and 8-oxodGuo eluted at different distinct positions and were able to be assayed without cross-contamination.

The genomic DNA was prepared from the kidneys obtained from the mice at different stages of growth, and their 8-oxodGuo content was determined. The values were expressed as numbers of 8-oxodGuo per 10^6^ residues of dGuo. To avoid the influence of age on the level of DNA oxidation, we compared the levels of 8-oxodGuo in db/db mice and their lean db/m littermates. These results are presented in Figure 3(b). The 8-oxodGuo content increased slightly with age, and the maximum values were observed at 16 weeks after birth, at which point the examination was terminated. This age-dependent increase in the 8-oxodGuo content was found in both types of mice. The rates of increase were insignificant in db/db mice compared with those in db/m mice.

The amounts of 8-oxoGuo were determined in RNA derived from kidneys of the two types of mice. Precautions were again taken to minimize the levels of oxidation of the materials during the preparation of RNA as well as the enzymatic digestion to nucleosides. Under the conditions used for liquid chromatography, Guo and 8-oxoGuo were eluted at distinct positions (Figure 3(c)). To ensure the accurate determination of these nucleosides, ^15^N-substituted compounds were included as internal standards.  Figure 3(d) shows the 8-oxoGuo contents of RNA derived from the kidneys of the two types of mice, and the values were analogously expressed as the numbers of 8-oxoGuo per 10^6^ residues of Guo. There were age-dependent increases in the 8-oxoGuo content in both the db/db and the db/m mice. However, the db/db mice showed higher levels of 8-oxoGuo than their lean db/m littermates, and the differences in the levels of 8-oxoGuo/10^6^ Guo in RNA between db/db and db/m mice were more apparent than those observed with 8-oxodGuo/10^6^ dGuo in DNA.

### 3.3. Quantification of Oxidized Guanine Nucleosides in Urine

**Figure 4:**
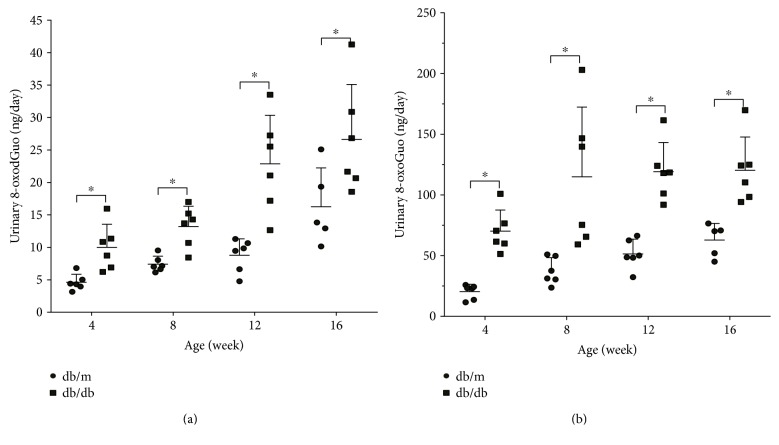
The HPLC-MS/MS determination of the concentrations of 8-oxodGuo and 8-oxoGuo in the urine of db/db mice. db/db represents BKS.Cg-Dock7^m^ +/+ Lepr^db^/JNju mice, and db/m represents their lean littermates. Samples from 4-, 8-, 12-, and 16-week-old mice were examined. (a) Increases in the level of 8-oxodGuo/day in the urine of db/db and db/m mice (*n* = 5 or 6 per group). (b) Increases in the level of 8-oxoGuo/day in the urine of db/db and db/m mice (*n* = 5 or 6 per group). The data are shown as the means ± SD. (black-colored dots) db/m. (black-colored squares) db/db, ^∗^*P* < 0.05 versus the respective controls.

The urine samples were analyzed by HPLC-MS/MS. [^15^N_5_]8-oxodGuo and [^15^N_2_^13^C_1_]8-oxoGuo were used as internal standards, and the values were expressed as the total volume per day. The results are shown in Figure 4. The amounts of 8-oxodGuo and 8-oxoGuo increased with age in both types of mice, and significant differences were observed between the db/db and their lean db/m littermates. The levels of 8-oxodGuo, as well as 8-oxoGuo, were much higher in the urine of db/db mice than in age-matched control db/m mice. Furthermore, in the urine, the level of 8-oxoGuo was significantly higher than that of 8-oxodGuo.

### 3.4. Correlations of 8-oxodGuo/8-oxoGuo between Urine and Tissue

**Figure 5:**
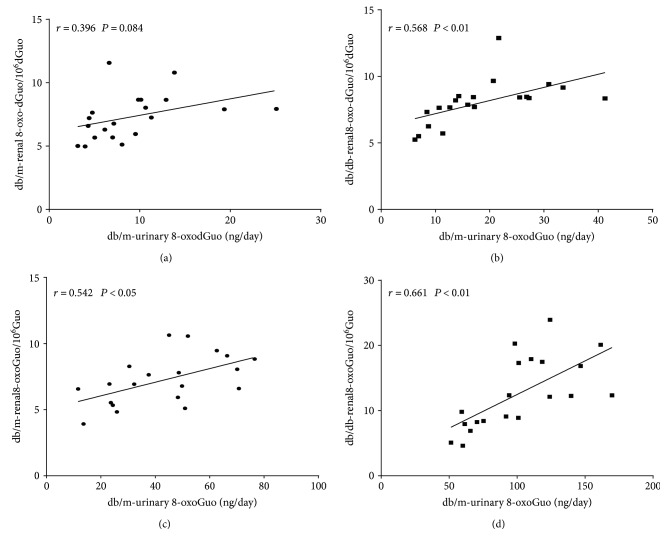
Correlations between urinary 8-oxodGuo/8-oxoGuo and renal 8-oxodGuo/8-oxoGuo. (a) Correlation between urinary 8-oxodGuo/day and renal 8-oxodGuo/10^6^dGuo in db/m mice (*r* = 0.396, *P* = 0.084). (b) Correlation between urinary 8-oxodGuo/day and renal 8-oxodGuo/10^6^dGuo in db/db mice (*r* = 0.568, *P* < 0.01). (c) Correlation between urinary 8-oxoGuo/day and renal 8-oxoGuo/10^6^Guo in db/m mice (*r* = 0.542, *P* < 0.05). (d) Correlation between urinary 8-oxoGuo/day and renal 8-oxoGuo/10^6^Guo in db/db mice (*r* = 0.661, *P* < 0.01).

Because urinary 8-oxodGuo or 8-oxoGuo are known as general oxidative markers, we analyzed the correlation of these markers between renal tissue and urine. As shown in Figure 5, urinary 8-oxodGuo and 8-oxoGuo were associated with their respective levels in the kidney, except for 8-oxodGuo in db/m mice (urinary 8-oxodGuo/day versus renal 8-oxodGuo/10^6^dGuo in db/m mice, *r* = 0.396, *P* = 0.084; urinary 8-oxodGuo/day versus renal 8-oxodGuo/10^6^dGuo in db/db mice, *r* = 0.568, *P* < 0.01; urinary 8-oxoGuo/day versus renal 8-oxoGuo/10^6^Guo in db/m mice, *r* = 0.542, *P* < 0.05; urinary 8-oxoGuo/day versus renal 8-oxoGuo/10^6^Guo in db/db mice *r* = 0.661, *P* < 0.01).

### 3.5. Correlations between 8-oxodGuo/ 8-oxoGuo and Urinary Micro-albumin

**Figure 6:**
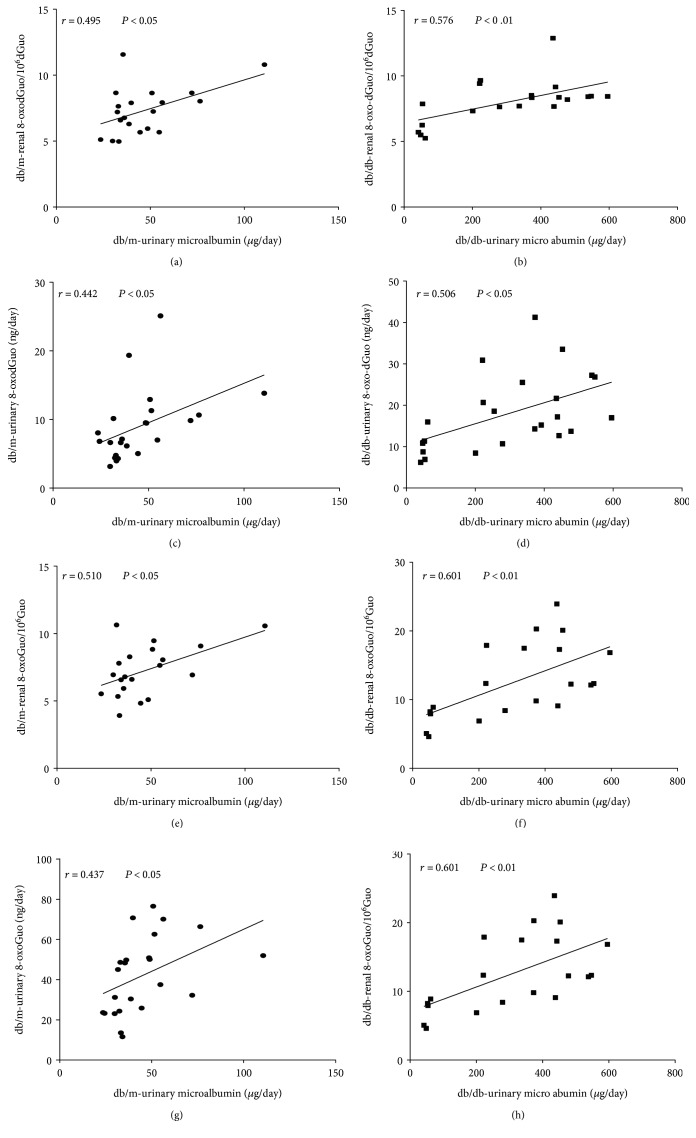
Correlations between 8-oxoGuo/ 8-oxodGuo and urinary microalbumin. (a) Correlation between renal 8-oxodGuo/10^6^dGuo and urinary micro-albumin/day in db/m mice (*r* = 0.495, *P* < 0.05). (b) Correlation between renal 8-oxodGuo/10^6^dGuo and urinary microalbumin/day in db/db mice (*r* = 0.576, *P* < 0.01). (c) Correlation between 8-oxodGuo and microalbumin per day in urine from db/m mice (*r* = 0.442, *P* < 0.05). (d) Correlation between 8-oxodGuo and micro-albumin per day in urine from db/db mice (*r* = 0.506, *P* < 0.05). (e) Correlation between renal 8-oxoGuo/10^6^Guo and urinary micro-albumin/day in db/m mice (*r* = 0.510, *P* < 0.05). (f) Correlation between renal 8-oxoGuo/10^6^Guo and urinary micro-albumin/day in db/db mice (*r* = 0.601, *P* < 0.01). (g) Correlation between 8-oxoGuo and micro-albumin per day in urine from db/m mice (*r* = 0.437, *P* < 0.05). (h) Correlation between 8-oxoGuo and microalbumin per day in urine from db/db mice (*r* = 0.701, *P* < 0.01).

Figure 6 shows the relationship between the levels of 8-oxodGuo or 8-oxoGuo and urinary microalbumin. 8-oxodGuo and 8-oxoGuo levels in kidney closely paralleled the increase in microalbumin levels in urine (renal 8-oxodGuo/10^6^dGuo versus urinary micro-albumin/day in db/m mice, *r* = 0.495, *P* < 0.05; renal 8-oxodGuo/10^6^dGuo and urinary microalbumin/day in db/db mice, *r* = 0.576, *P* < 0.01; renal 8-oxoGuo/10^6^Guo versus urinary microalbumin/day in db/m mice, *r* = 0.510, *P* < 0.05; renal 8-oxoGuo/10^6^Guo versus urinary microalbumin/day in db/db mice, *r* = 0.601, *P* < 0.01). There were also significant correlations between 8-oxodGuo or 8-oxoGuo and microalbumin in urine (8-oxodGuo versus microalbumin per day in urine from db/m mice, *r* = 0.442, *P* < 0.05; 8-oxodGuo versus microalbumin per day in urine from db/db mice, *r* = 0.506, *P* < 0.05; 8-oxoGuo versus microalbumin per day in urine from db/m mice, *r* = 0.437, *P* < 0.05; 8-oxoGuo versus microalbumin per day in urine from db/db mice, *r* = 0.701, *P* < 0.01). Multiple linear regression analysis of microalbumin and nucleotide acid oxidation markers identified urinary 8-oxoGuo as the predominant indicator of the severity of microalbuminuria (Supplementary Table 4s).

## 4. Discussion

DNA and RNA oxidations have been linked to diseases such as cancer, arteriosclerosis, neurodegeneration, and diabetes. The measurement of urinary 8-oxodGuo and 8-oxoGuo has accordingly gained increasing interest, probably because such measurement constitutes a noninvasive method that can be used *in vivo* in humans as well as animals. A previous study provided evidence that 8-oxodGuo in urine is a useful clinical marker for predicting the development of diabetic nephropathy in diabetic patients [6]. However, another study recently found that, compared with UACR, urinary 8-OHdGuo is not a useful clinical marker for predicting such an outcome [8]. Given these conflicting findings, we believe that readers should pay attention to the following points: First, the nomenclature used is not consistent across authors, and the abbreviations for the same chemical entity differ over time and among authors, which may confuse readers and cause them to draw a different conclusion. Second, various methods have been used to determine the levels of 8-oxodGuo, and historically, the quantitation of urinary 8-oxodGuo in diabetic nephropathy research has mainly been based on HPLC with electrochemical detection (HPLC-ECD) or ELISA [5, 6, 20–22], methods that are hampered by insufficient specificity and sensitivity [23].

Given that 2-deoxyguanosine (DNA) is oxidized to form 8-oxodGuo while guanosine (RNA) is oxidized to form 8-oxoGuo, this study use 8-oxodGuo and 8-oxoGuo for the oxidized guanine nucleosides from DNA and RNA, respectively. We used a procedure based on HPLC-MS/MS, a gold-standard technique meeting the European Communities requirement, to determine the oxidative damage to DNA and RNA in renal tissue and urine samples more accurately. Although we used db/db mice of different ages to investigate changes in the oxidative guanine nucleosides aggravation of renal injury in the early stage of diabetic nephropathy, we compared the levels of oxidative guanine nucleosides in db/db mice and their lean db/m littermates to avoid any influence of age on the level of nucleoside oxidation.

Of note, the db/db mice showed higher levels of renal 8-oxodGuo and 8-oxoGuo than their lean db/m littermates. However, a detailed statistical analysis of db/db mice and their lean db/m littermates showed that only the rates of increased renal 8-oxoGuo were significant in db/db mice to db/m mice. Db/db mice showed higher levels of renal 8-oxoGuo than their lean db/m littermates. These results not only indicate that db/db mice with early symptoms of diabetic nephropathy suffered from more extensive oxidative nucleoside damage than their lean littermate control mice but also confirm our findings that RNA oxidation appeared earlier and was more apparent than DNA oxidation in diabetic nephropathy [13]. Though increased RNA oxidation in renal tissue is difficult and cannot alone be used to predictive for development diabetic nephropathy *in vivo* [24], it may provide an important hint that RNA oxidation is involved in the development of diabetic nephropathy.

The quantification of oxidized guanine nucleosides in urine also revealed markedly higher levels of 8-oxodGuo, as well as 8-oxoGuo, in the urine of db/db mice compared with age-matched control db/m mice, and the level of 8-oxoGuo was significantly higher than that of 8-oxodGuo. However, the urinary excretion of 8-oxodGuo and 8-oxoGuo is thought to indicate global oxidative stress to DNA or RNA and is recognized as a general marker of DNA and RNA oxidation [25–27]. Therefore, it is important to determine whether or not urinary 8-oxodGuo and 8-oxoGuo are associated with the same oxidative damage in renal tissue of diabetic nephropathy. Interestingly, our examination of the relationship of 8-oxodGuo and 8-oxoGuo between urine and renal tissue showed that the amount of 8-oxodGuo and 8-oxoGuo in urine was in good agreement with the levels of renal 8-oxodGuo/10^6^dGuo and 8-oxoGuo/10^6^Guo, respectively. The combination of the above parameters with urinary 8-oxodGuo is a confirmed and widely investigated biomarker of diabetic nephropathy, and the ratios of 8-oxoGuo to 8-oxodGuo in renal tissue and urine were considerably high in db/db mice. We therefore propose that urinary 8-oxoGuo may also be a novel, noninvasive, and easily detected biomarker of DKD. However, caution should be practiced when interpreting these results, as our study was unable to differentiate the causal relationship of 8-oxodGuo and 8-oxoGuo between urine and renal tissue.

It is very important to emphasize the early identification, prevention, and treatment of DKD, since individuals with DKD are at significant risk of progression to end-stage renal disease (ESRD) and cardiovascular morbidity and mortality [28]. Reactive oxygen species (ROS) production in diabetes may be a common pathway linking diverse pathogenic mechanisms of diabetic vascular complications. Therefore, the assessment of oxidative stress in diabetic patients may be important for the prediction and prevention of diabetic complications. To determine whether or not DNA/RNA oxidation is related to the early stage of renal lesions in diabetic nephropathy, we further investigated the relationship between urinary microalbumin and 8-oxodGuo or 8-oxoGuo in the kidney or urine. Renal 8-oxodGuo or 8-oxoGuo levels were found to be closely related to the increase in urinary microalbumin levels, and we also noted significant correlations between 8-oxodGuo or 8-oxoGuo and microalbumin in urine. Furthermore, the increases in 8-oxodGuo or 8-oxoGuo paralleled the aggravation of diabetic nephropathy in db/db mice at 4 to 16 weeks of age.

Most previous investigations have analyzed the relationship between 8-oxodGuo, a marker of DNA oxidation, and DKD [5, 6, 20–22], even though RNA is known to be more prone to oxidation than DNA. The low level of interest in RNA oxidation over the years is likely the reason why RNA oxidation has only recently being recognized as a disease-relevant mechanism [29]. In fact, the oxidation of RNA is minimally determined by genetic factors which may leave ample room for therapeutic interventions [29, 30]. Oxidized RNA is recently found in a large variety of diseases and is viewed as an early event in some diseases [9, 10]. Broedbaek et al. found that urinary 8-oxoGuo excretion could predict overall death and death from complications in type 2 diabetes, while urinary 8-oxodGuo could not [11, 31]. Our previous study also indicated that there were only 8-oxoGuo that had significant differences between diabetes patients with complications and those without complications [12]. However, the utility of urinary 8-oxoGuo as an indicator reflecting oxidative damage in the kidney remains unclear.

The present study showed that 8-oxoGuo was more closely related to the levels of urinary microalbumin and early pathological changes of diabetic nephropathy than 8-oxodGuo. A glomerular ultrastructure examination showed that podocyte effacement was more apparent in 4-week-old db/db mice than in their lean littermates. However, only the levels of 8-oxoGuo in the kidney were elevated in 4-week-old db/db mice compared with their lean littermates. Although 4-week-old db/db mice showed greater amounts of urinary 8-oxodGuo and 8-oxoGuo than controls, the elevation of 8-oxoGuo in the urine and kidney was greater and appeared earlier than that of 8-oxodGuo, especially in the renal tissue. Multiple linear regression analysis of microalbumin and nucleotide acid oxidation markers also identified urinary 8-oxoGuo as the predominant indicator of the severity of microalbuminuria. These results indicate that RNA oxidation is more apparent and more closely related to early diabetic nephropathy than DNA oxidation. If the source of urinary 8-oxoGuo could be clarified, it might provide new insight into the pathogenesis of diabetic nephropathy. Further studies are needed to investigate the mechanisms of RNA oxidation in the development of DKD and confirm them in large population studies.

## 5. Conclusion

The present study showed that RNA oxidation occurs earlier and is more closely related to early diabetic nephropathy than DNA oxidation in db/db mice. Urinary 8-oxoGuo may represent a novel, noninvasive, and easily detected biomarker of diabetic kidney diseases if these results could be confirmed in large population studies. Oxidative damage of RNA may provide new insight into the pathogenesis of diabetic nephropathy. Further studies should investigate the mechanism underlying the association between RNA oxidation and diabetic nephropathy.

## Supplementary Material

Supplementary Table 1s Optimized measurement conditions of DNA from kidney. Supplementary Table 2s Optimized measurement conditions of RNA from kidney. Supplementary Table 3s Optimized measurement conditions of urinary 8-oxoGuo and 8-oxodGuo. Supplementary Table 4s Multiple linear regression analysis between urinary micro-albuminuria and nucleotide acid oxidation markers.

## Figures and Tables

**Table 1:** Biochemical and physical characteristics of experimental groups.

Characteristic	db/m—4 w	db/db—4 w	db/m—8 w	db/db—8 w	db/m—12 w	db/db—12 w	db/m—16 w	db/db—16 w
Body weight (g)	15.5 ± 1.4	23.8 ± 0.8	23.8 ± 1.4	45.7 ± 2.1	26.7 ± 1.3	48.9 ± 5.0	28.9 ± 2.6	44.5 ± 3.5
Kidney weight (mg)	142.3 ± 8.8	173.6 ± 29.7	176.2 ± 17.9	238.7 ± 67.6	174.3 ± 13.4	234 ± 26.4	186.8 ± 25.7	222.7 ± 40.6
Kidney wt/body wt, ×10^−3^	8.0 ± 0.5	5.3 ± 1.0	7.8 ± 0.7	5.2 ± 1.3	7.5 ± 0.5	4.9 ± 0.6	7.6 ± 0.8	5.5 ± 1.1
Blood glucose (mmol/L)	7.9 ± 0.5	13.4 ± 3.9	8.1 ± 0.8	24.1 ± 5.5	6.8 ± 0.6	29.7 ± 2.9	6.8 ± 0.3	34.7 ± 3.2
Plasma creatinine (mg/dL)	0.094 ± 0.007	0.083 ± 0.01	0.088 ± 0.01	0.089 ± 0.01	0.093 ± 0.005	0.103 ± 0.02	0.096 ± 0.008	0.106 ± 0.01
