# Supplementary material for: Increased Oxidative Damage of RNA in Early-Stage Nephropathy in db/db Mice
Source: Oxid Med Cell Longev. 2017 Oct 19;2017:2353729. doi: 10.1155/2017/2353729 (PMC5671745; doi:10.1155/2017/2353729)
Supplement: Supplementary file 1 — Supplementary Table 1s Optimized measurement conditions of DNA from kidney. Supplementary Table 2s Optimized measurement conditions of RNA from kidney. Supplementary Table 3s Optimized measurement conditions of urinary 8-oxoGuo and 8-oxodGuo. Supplementary Table 4s Multiple linear regression analysis between urinary micro-albuminuria and nucleotide acid oxidation markers. [file 2353729.f1.docx]

**Supplementary Table 1s Optimized measurement conditions of DNA from kidney**

| Compound Name | ISTD? | Precursor Ion | MS1 Res | Product Ion | MS2 Res | Dwell | Fragmentor | Collision Energy | Cell Accelerator Voltage | Polarity |
| --- | --- | --- | --- | --- | --- | --- | --- | --- | --- | --- |
| 8-oxodGuo-ISTD | **√** | 289 | Unit | 173 | Unit | 100 | 380 | 15 | 3 | Positive |
| 8-oxodGuo |  | 284 | Unit | 168 | Unit | 100 | 380 | 8 | 3 | Positive |
| 8-oxodGuo |  | 284 | Unit | 140 | Unit | 100 | 380 | 40 | 3 | Positive |
| dGuo-IS | **√** | 273 | Unit | 157 | Unit | 100 | 380 | 9 | 3 | Positive |
| dGuo |  | 268 | Unit | 152 | Unit | 100 | 380 | 40 | 3 | Positive |
| dGuo |  | 268 | Unit | 135 | Unit | 100 | 380 | 40 | 3 | Positive |

**Supplementary Table 2s Optimized measurement conditions of RNA from kidney**

| Compound Name | ISTD? | Precursor Ion | MS1 Res | Product Ion | MS2 Res | Dwell | Fragmentor | Collision Energy | Cell Accelerator Voltage | Polarity |
| --- | --- | --- | --- | --- | --- | --- | --- | --- | --- | --- |
| 8-oxoGuo-ISTD | **√** | 303 | Unit | 171 | Unit | 100 | 380 | 12 | 3 | Positive |
| 8-oxoGuo |  | 300 | Unit | 168 | Unit | 100 | 380 | 14 | 3 | Positive |
| 8-oxoGuo |  | 300 | Unit | 140 | Unit | 100 | 380 | 38 | 3 | Positive |
| Guo-IS | **√** | 289 | Unit | 157 | Unit | 100 | 380 | 12 | 3 | Positive |
| Guo |  | 284 | Unit | 152 | Unit | 100 | 380 | 40 | 3 | Positive |
| Guo |  | 284 | Unit | 135 | Unit | 100 | 380 | 42 | 3 | Positive |

**Supplementary Table 3s Optimized measurement conditions of urinary 8-oxoGuo and 8-oxodGuo**

| Compound Name | ISTD? | Precursor Ion | MS1 Res | Product Ion | MS2 Res | Dwell | Fragmentor | Collision Energy | Cell Accelerator Voltage | Polarity |
| --- | --- | --- | --- | --- | --- | --- | --- | --- | --- | --- |
| 8-oxoGuo-ISTD | **√** | 303 | Unit | 171 | Unit | 100 | 380 | 12 | 3 | Positive |
| 8-oxoGuo |  | 300 | Unit | 168 | Unit | 100 | 380 | 14 | 3 | Positive |
| 8-oxoGuo |  | 300 | Unit | 140 | Unit | 100 | 380 | 38 | 3 | Positive |
| 8-oxodGuo-ISTD | **√** | 289 | Unit | 173 | Unit | 100 | 380 | 10 | 3 | Positive |
| 8-oxodGuo |  | 284 | Unit | 168 | Unit | 100 | 380 | 10 | 3 | Positive |
| 8-oxodGuo |  | 284 | Unit | 140 | Unit | 100 | 380 | 32 | 3 | Positive |

**Supplementary Table 4s Multiple linear regression analysis between urinary micro-albuminuria and nucleotide acid oxidation markers.**

| Variable | Unstandardized Coefficients | | Standardized Coefficients | | T | Sig. |
| --- | --- | --- | --- | --- | --- | --- |
|  | **B** | **Std. Error** | **Beta** | |  |  |
| Constant | -139.098 | 65.701 |  | -2.117 | | .041 |
| renal 8-oxoGuo/10^6^Guo | 13.893 | 4.880 | .370 | 2.847 | | .007 |
| renal 8-oxodGuo/10^6^dGuo | -3.115 | 10.101 | -.029 | -.308 | | .760 |
| Urinary 8-oxoGuo | 3.305 | .510 | .723 | 6.480 | | .000 |
| Urinary 8-oxodGuo | -2.498 | 2.704 | -.122 | -.924 | | .362 |

Dependent Variable: urinary micro-albumin
